# Is screen time associated with anxiety or depression in young people? Results from a UK birth cohort

**DOI:** 10.1186/s12889-018-6321-9

**Published:** 2019-01-17

**Authors:** Jasmine N. Khouja, Marcus R. Munafò, Kate Tilling, Nicola J. Wiles, Carol Joinson, Peter J. Etchells, Ann John, Fiona M. Hayes, Suzanne H. Gage, Rosie P. Cornish

**Affiliations:** 1 MRC Integrative Epidemiology Unit, University of Bristol, Bristol, UK; 2 UK Centre for Tobacco and Alcohol Studies and School of Psychological Science, University of Bristol, Bristol, UK; 3 Bristol Medical School: Population Health Sciences, University of Bristol, Bristol, UK; 4 College of Liberal Arts, Bath Spa University, Bath, UK; 5 Swansea University Medical School, Swansea, UK; 6 Department of Psychological Sciences, University of Liverpool, Liverpool, UK; 7 University of Bristol Students’ Health Service, Bristol, UK

**Keywords:** Screen time, Anxiety, Depression, Mental health, ALSPAC, Is screen time associated with anxiety or depression in young people? Results from a UK birth cohort

## Abstract

**Background:**

There is limited and conflicting evidence for associations between use of screen-based technology and anxiety and depression in young people. We examined associations between screen time measured at 16 years and anxiety and depression at 18.

**Methods:**

Participants (*n* = 14,665; complete cases *n =* 1869) were from the Avon Longitudinal Study of Parents and Children, a UK-based prospective cohort study. We assessed associations between various types of screen time (watching television, using a computer, and texting, all measured via questionnaire at 16y), both on weekdays and at weekends, and anxiety and depression (measured via the Revised Clinical Interview Schedule at 18y). Using ordinal logistic regression, we adjusted for multiple confounders, particularly focussing on activities that might have been replaced by screen time (for example exercising or playing outdoors).

**Results:**

More time spent using a computer on weekdays was associated with a small increased risk of anxiety (OR for 1–2 h = 1.17, 95% CI: 1.01 to 1.35; OR for 3+ hours = 1.30, 95% CI: 1.10 to 1.55, both compared to < 1 h, *p* for linear trend = 0.003). We found a similar association between computer use at weekends and anxiety (OR for 1–2 h = 1.17, 95% CI: 0.94 to 1.46; OR for 3+ hours = 1.28, 95% CI: 1.03 to 1.48, *p* for linear trend = 0.03). Greater time spent using a computer on weekend days only was associated with a small increased risk in depression (OR for 1–2 h = 1.12, 95% CI: 0.93 to 1.35; OR for 3+ hours = 1.35, 95% CI: 1.10 to 1.65, *p* for linear trend = 0.003). Adjusting for time spent alone attenuated effects for anxiety but not depression. There was little evidence for associations with texting or watching television.

**Conclusions:**

We found associations between increased screen time, particularly computer use, and a small increased risk of anxiety and depression. Time spent alone was found to attenuate some associations, and further research should explore this.

**Electronic supplementary material:**

The online version of this article (10.1186/s12889-018-6321-9) contains supplementary material, which is available to authorized users.

## Background

The amount and nature of time spent using screen-based devices such as televisions, computers, and mobile phones has changed over recent years. A report in 2017 suggested that British children aged 5–15 years spent 1.5 more hours per week online than watching TV which is in contrast to their findings in 2007 when they spent roughly 5 h more per week watching TV than online [1]. Patterns of screen use also differ depending on time of the week, with more time spent using screens on weekends than weekdays [2]. The report found that screen-based products were commonly used by children and adolescents, with 79% of 12–15 year olds owning their own smart phone, and 48% of 5–15 year olds having a TV in their bedroom in 2016 [1]. Alongside increases in screen time there has been an increase in the recorded incidence of common mental health disorders in children and adolescents [3], leading us to question whether they are related.

Teychenne and colleagues [4] recently systematically reviewed the literature on the association between sedentary behaviour and anxiety; they included studies that specifically examined screen time. Of the four studies in the review that explored the association between increased screen time and anxiety, two found positive associations [5, 6]. However, like many in this field, these studies were cross-sectional and could not assess the temporal direction of association. The two remaining studies either found no association [7] or an inverse association [8] (in a cross-sectional study and prospective cohort, respectively). Of the four studies, only one [5] was assessed as having strong methodological quality. Other reviews of the literature concluded that there was insufficient or inconclusive evidence for an association between screen time and anxiety [9, 10].

There is more consistent evidence for an association between screen time and depression [11–13]. However the evidence base is still limited, with research conclusions restricted by methodological limitations such as cross-sectional designs and broad age ranges (including both children and adults) [4]. What evidence there is indicates that associations between screen time and depression may operate in both directions [12, 14].

We therefore examined the association between screen time and both anxiety and depression during adolescence using prospectively collected longitudinal data from the Avon Longitudinal Study of Parents and Children (ALSPAC) [15, 16]. Building on previous research, ours is the first study to assess the association between screen time (and different types of screen time) in a prospective UK cohort. Importantly, we also attempted to adjust for a range of other activities in order to identify what other activities are sacrificed for screen time. Such measures include time spent outside, time spent socialising, and time spent alone. We also separately investigated the associations with weekday and weekend screen use.

## Methods

### Participants and recruitment

**Fig. 1:**
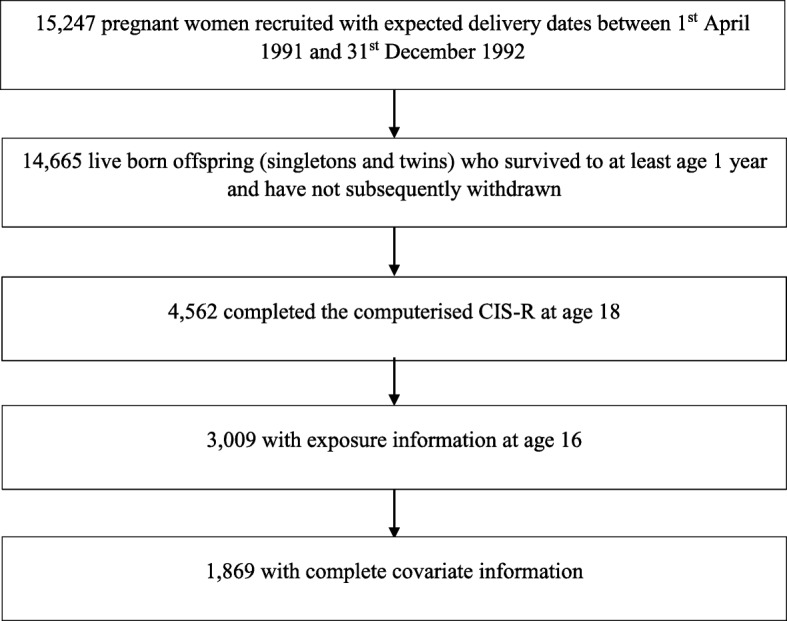
Flow diagram depicting the children from the ALSPAC cohort included in the present study

ALSPAC is a large prospective cohort which initially recruited 14,541 pregnant mothers living in and around Bristol, England, and due to give birth between 1st April 1991 and 31st December 1992. Of the 14,062 live births, 13,988 children were alive at 1 year. A further 706 pregnant women – individuals who were eligible but failed to enrol in the original recruitment phase – were recruited in subsequent years. This cohort has been described in detail previously [15, 16]. The study website contains details of all available data through a fully searchable data dictionary [17]. The sample in this study consists of the 14,665 singletons and twins alive at one year who had not subsequently withdrawn from the study (Fig. 1).

### Ethics statement

Ethics approval for the study was obtained from the ALSPAC Ethics and Law Committee and the Local Research Ethics Committee (NHS North Somerset & South Bristol Research Ethics Committee). Full details of ethics committee approval references for ALSPAC can be found online (http://www.bristol.ac.uk/alspac/researchers/research-ethics/). This study was approved by the ALSPAC Executive Committee. Study participants who complete questionnaires consent to the use of their data by approved researchers. Up until age 18 an overarching parental consent was used to indicate parents were happy for their child (the study participant) to take part in ALSPAC. Consent for data collection and use was implied via the written completion and return of questionnaires. Study participants have the right to withdraw their consent for specific elements of the study, or from the study as a whole, at any time.

### Measures

#### Screen time use

Screen time was assessed in a study questionnaire administered when the children were aged 16 years. Respondents were asked six questions relating to watching television, computer use, and texting (Additional file 1, section 1). Answers were categorised as less than one hour, one to two hours, and three or more hours per average day and separate responses were collected for weekend and weekday use.

#### Anxiety and depression

Anxiety and depression were measured at approximately 18 years, using a self-administered, computerised version of the revised Clinical Interview Schedule (CIS-R) [18] completed during a study clinic. The CIS-R asks questions about a range of symptoms and can be used to assign ICD-10 diagnoses of depression and anxiety disorders [19, 20]. Anxiety and depression were coded as three-level variables categorised as: no anxiety/depression; symptoms but no diagnosis; and diagnosis. For anxiety, symptoms related to general anxiety, phobias, panic and worry; for depression, symptoms related to depression or depressive thoughts. Sleep, concentration and fatigue scores were not used to indicate symptoms of depression due to their lack of specificity [21–23]. Earlier depression and anxiety at 7, 10, 13 and 15 years were assessed using the Development and Well-Being Assessment (DAWBA) [24]. At 7, 10 and 13 years, computerised DAWBA questions were completed by the parent of the child, at 15 years the computerised DAWBA questionnaire was self-administered. A computerised algorithm was used to derive ordered categorical variables (with 6 categories) for anxiety and depression, with higher categories indicating increasing levels of symptoms [24]. Due to small numbers in some of the categories, anxiety and depression at 7, 10 and 13 were dichotomised into low (categories 1 and 2) and medium/high (categories 3 to 6); anxiety and depression at 15 were regrouped into low (categories 1 and 2), medium (category 3) and high (categories 4–6).

#### Potential confounders

Previous literature was examined to select potential confounders. These included sex and anxiety/depression measured at age 15 years. Parental covariates were: maternal age at delivery; maternal anxiety measured via questionnaire at seven time points on and after the child was 8 months (these were used to create a single binary variable – maternal anxiety – which was positive if the mother reported anxiety at any time point, no if she reported no anxiety on all occasions, and missing otherwise); maternal depression measured when the child was 8 months using the Edinburgh Post-natal Depression Scale (EPDS) [25]; maternal education measured during pregnancy and determined by the mother’s highest educational qualification (a 4-level categorical variable, Additional file 1, section 1); and parental socio-economic status (SES). SES was measured when the mothers were 32 weeks pregnant and was based on the higher of the mother or partner’s occupational social class, dichotomised into non-manual (professional, managerial or skilled professions) and manual (partly or unskilled occupations).

Childhood covariates included for further adjustment were: IQ, measured at 8 years using the Wechsler Intelligence Scale for children (WISC-III^UK^) [26]; parental conflict measured at 8 months; presence of the child’s father in the child’s home measured at 4 years; number of people living in the child’s home measured at 4 years; bullying measured at 16 years; and early family TV use measured at 18 months.

We also adjusted for covariates relating to time spent doing other activities: exercising; on transport; playing outdoors in summer and winter; playing with others; making, drawing and constructing things; being alone; completing home or college work; reading; playing musical instruments; talking on a mobile; and talking on a landline phone.

Further details about these measures including the measurement methods and definitions are available in the supplementary material (Additional file 1, section 1).

### Data analysis

Only 1869 participants (12.7% of the overall study sample) had complete data on the outcomes, exposure and covariates, so we used multiple imputation (MI) using chained equations (fully conditional specification) [27] to address missing data. Logistic regression was used to assess whether earlier depression and anxiety (at 7 years) was associated with missing outcome information after adjustment for covariates to assess whether the outcome data were likely to be missing not at random (MNAR) conditional on the baseline covariates.

The MI models, in which 100 datasets were imputed, included the exposures, outcomes and all covariates listed above as well as auxiliary variables – included to make the missing at random assumption more plausible. These variables included all the earlier measures of depression and anxiety as well as earlier measures of screen use and other activities and additional measures predictive of childhood and parental factors. Further details of the imputation models, including the auxiliary variables, are given in the supplementary material (Additional file 1, section 2 and Table S1).

We assessed the association between screen time, separately for types of device (watching television, computer use, and texting) and timing (weekday or weekend), and anxiety and depression using ordinal logistic regression models. This gave an odds ratio for being in a higher anxiety/depression category for a one unit change in a covariate. The ordinal logistic regression model assumes that the relationship between the lowest category of the outcome and all the higher categories are equal to the relationship between the second lowest category and all the higher categories; a Brant test was conducted to confirm the data did not violate this assumption [28]. Covariates were grouped and added to the unadjusted model (model 1) to examine their effect on the association. Model 2 adjusted for sex, maternal age, anxiety/depression at 15 years, maternal anxiety and depression, maternal education, and parental SES. Model 3 also included IQ, parental conflict, presence of the child’s father, number of people living in the child’s home, bullying, and family TV use in early life. Each of the sub-models of model 4 additionally adjusted for time spent engaging in one other activity on weekdays or weekends (time alone [model 4a], on transport [model 4b], playing outdoors in summer [model 4c], playing outdoors in winter [model 4d], playing with others [model 4e], drawing, making or constructing things [model 4f], exercising [model 4 g], completing school or college work [model 4 h], reading [model 4i], playing musical instruments [model 4j], talking on a mobile phone [model 4 k] and talking on a landline phone [model 4 l]). *P*-values for the association between types of screen time and anxiety and depression were obtained using a test for linear trend.

We carried out the following sensitivity analyses. Firstly we repeated the above analyses for the complete case sample (*n* = 1869). Second, because there was evidence that individuals with missing data were more likely to have higher levels of anxiety/depression we carried out a sensitivity analysis in which all individuals with imputed anxiety/depression were re-categorised as one level higher than predicted in each imputed dataset (except when they were already predicted as being in the highest category).

All analyses were carried out in Stata (versions 14 and 15) (Stata Corp LP, College Station, TX USA); MI used the mi impute command.

## Results

### Study sample

Of the 14,665 participants in the study, 4562 (31.1%) had completed the CIS-R questions relating to anxiety and depression at age 18, of whom 3109 (68.1%) had also completed the questionnaire at 16 regarding screen time. There were 1869 individuals with complete covariate information. This information is summarised in Fig. 1. Characteristics of the complete cases and the 14,665 individuals included in this study are given in Table 1. Characteristics were similar among the complete cases and the individuals included, however those with complete data were more likely to be female, have an older mother with a higher level of education and have a mother with maternal anxiety (Table 1). Similar patterns of screen use, anxiety and depression were seen in both the complete cases and the included individuals (Table 1).

**Table 1:** Characteristics of the ALSPAC-enrolled sample and complete cases

		Enrolled singletons and twins, alive at 1 year and not withdrawn from the study (n = 14,665)^1^	Complete cases (*n* = 1869)
Sex	Male Female	7524 (51%) 7141 (49%)	802 (43%) 1067 (57%)
Maternal age	< 25 25–29 30+	3336 (24%) 5394 (39%) 5224 (37%)	173 (9%) 694 (37%) 1002 (54%)
Mother’s education^2^	CSE/vocational O level A level Degree/higher	3723 (30%) 4287 (35%) 2786 (22%) 1599 (13%)	237 (13%) 620 (33%) 570 (31%) 442 (24%)
Family occupational social^3^ class	Non-manual Manual	9254 (81%) 2231 (19%)	1698 (91%) 171 (9%)
Maternal anxiety	No Yes	4808 (51%) 4584 (49%)	1158 (62%) 711 (38%)
Biological father lives with child (age 4)	No Yes	1217 (13%) 8270 (87%)	123 (7%) 1746 (93%)
Parental conflict	No Yes	10,008 (88%) 1305 (12%)	1719 (92%) 150 (8%)
Child bullied since age 12 (at age 16)	No Yes	4175 (83%) 885 (18%)	1549 (83%) 320 (17%)
Child IQ (age 8)	Mean (SD)	104 (17)^5^	110 (15)
Maternal depression (EPDS)^4^	Mean (SD)	5 (5)^6^	5 (4)
Number living in home	Median (IQR)	4 (4–5)^7^	4 (4–4)
Early family TV use score	Mean (SD)	4 (2)^8^	3 (2)
TV, weekdays	None/< 1 h 1–2 h 3+ hours	1306 (26%) 2648 (52%) 1118 (22%)	475 (25%) 1030 (55%) 364 (19%)
Texting, weekdays	None/< 1 h 1–2 h 3+ hours	2990 (59%) 1177 (23%) 898 (18%)	1199 (64%) 404 (22%) 266 (14%)
Computer use, weekdays	None/< 1 h 1–2 h 3+ hours	1171 (23%) 2416 (48%) 1481 (29%)	421 (23%) 928 (50%) 520 (28%)
TV, weekends	None/< 1 h 1–2 h 3+ hours	1015 (21%) 2270 (46%) 1615 (33%)	359 (19%) 914 (49%) 596 (32%)
Texting, weekends	None/< 1 h 1–2 h 3+ hours	2689 (55%) 1188 (24%) 1024 (21%)	1104 (59%) 436 (23%) 329 (18%)
Computer use, weekends	None/< 1 h 1–2 h 3+ hours	1108 (23%) 1950 (40%) 1843 (38%)	388 (21%) 770 (41%) 711 (38%)
Anxiety	No Symptoms Diagnosis	2410 (53%) 1630 (36%) 522 (11%)	1000 (54%) 686 (37%) 183 (10%)
Depression	No Symptoms Diagnosis	2736 (60%) 1466 (32%) 360 (8%)	1143 (61%) 598 (32%) 128 (7%)

^1^Denominators vary because the variables come from different questionnaires and have different completion rates.

^2^CSEs (Certificate of Secondary Education)/ and O levels were qualifications taken at age 16 – now replaced by GCSEs (General Certificate of Secondary Education) in England, Wales and Northern Ireland. A levels are exams taken at age 18 in these countries.

^3^Family occupational social class was based on the higher of the mother or partner’s occupational social class using the 1991 British Office of Population and Census Statistics (OPCS) classification and was dichotomized into non-manual (professional, managerial or skilled professions) and manual (partly or unskilled occupations).

^4^Edinburgh Post-natal Depression Scale (EPDS) [25]

^5^*N* = 7341

^6^*N* = 11,173

^7^*N* = 9472

^8^*N* = 10,654

Both anxiety and depression measured at age 7 years were associated with non-response at age 18 (results not shown): individuals with evidence of anxiety and depression at age 7 were more likely to have missing outcome data at age 18, suggesting that the outcomes could be MNAR, or MAR conditional on anxiety and depression at age 7 (we acknowledge that this cannot be determined from the observed data).

Among the 4562 adolescents who completed the CIS-R, 522 (11%) met the criteria for a diagnosis of anxiety and 360 (8%) met the criteria for a diagnosis of depression. 1630 (35%) displayed anxiety symptoms but did not meet the criteria for a diagnosis and 1466 (32%) displayed symptoms of depression but did not meet the criteria for a diagnosis (Table 1). These figures were slightly lower among those with complete covariate information (10 and 7% for diagnosis of anxiety and depression, respectively, and 37 and 32% for symptoms of anxiety and depression, respectively). In contrast, in the multiply imputed datasets, which took account of the fact that individuals with anxiety and depression were less likely to complete the CIS-R, the proportions with a diagnosis were higher, though this was not the case for symptoms (12 and 9% for diagnosis of anxiety and depression, respectively; 33 and 32% for symptoms of anxiety and depression, respectively). Screen time was slightly higher at weekends than on weekdays. This finding was consistent across all the types of device: 52% reported watching television for 1–2 h and 22% for 3 or more hours on weekdays compared to 46 and 33% (respectively) at weekends. The corresponding figures for computer use were 48 and 29% on weekdays and 40 and 38% at weekends; and for texting 23 and 18% on weekdays and 24 and 21% at weekends (Table 1).

### Anxiety

Table 2 shows that, after adjusting for confounders, there was no clear evidence of an association between time spent watching television or texting at age 16 and anxiety. There was moderate evidence for a small positive association between time spent using a computer on a weekday at age 16 and anxiety (OR for 1–2 h = 1.17, 95% CI: 1.01 to 1.35; OR for 3+ hours = 1.30, 95% CI 1.10 to 1.55, *p* for linear trend = 0.003, model 3; Table 2). However, this association attenuated to the null after adjusting for time spent alone (OR for 1–2 h = 1.12, 95% CI: 0.97 to 1.30; OR for 3+ hours = 1.14, 95% CI: 0.97 to 1.35, *p* for linear trend = 0.13; Table 2). Similarly, for weekend computer use, there was evidence of an association with anxiety at 18 years (OR for 1–2 h = 1.17, 95% CI: 0.94 to 1.46; OR for 3+ hours = 1.28, 95% CI: 1.03 to 1.58, *p* for linear trend = 0.03, model 3). Again, this association attenuated to the null after adjusting for time spent alone (OR for 1–2 h = 1.13, 95% CI: 0.91 to 1.41; OR for 3+ hours = 1.15, 95% CI: 0.92 to 1.44, *p* for linear trend = 0.25, Table 2). Adjustment for other activities (apart from time spent alone) had very little effect on the odds ratios (Additional file 1, Tables S2-S4).

**Table 2:** Odds ratios for associations between anxiety and watching television, computer use and texting (*n* = 14,665).

		Week days			Weekend days		
Model	Hours of use	OR	95%CI	*p*-value	OR	95%CI	*p*-value
Television							
1	< 1	1.00			1.00		
	1–2	0.95	0.83, 1.08		1.02	0.86, 1.21	
	3+	1.05	0.86, 1.27	0.63	1.06	0.88, 1.28	0.49
2	< 1	1.00			1.00		
	1–2	1.00	0.86, 1.17		1.07	0.89, 1.28	
	3+	1.08	0.89, 1.32	0.43	1.09	0.89, 1.32	0.42
3	< 1	1.00			1.00		
	1–2	1.02	0.87, 1.19		1.07	0.89, 1.28	
	3+	1.12	0.91, 1.37	0.27	1.09	0.90, 1.32	0.40
4a	< 1	1.00			1.00		
	1–2	1.03	0.88, 1.21		1.04	0.87, 1.23	
	3+	1.07	0.87, 1.32	0.50	1.02	0.84, 1.23	0.90
Computer use							
1	< 1	1.00			1.00		
	1–2	1.11	0.98, 1.26		1.05	0.86, 1.23	
	3+	1.26	1.05, 1.49	0.02	1.18	0.97, 1.44	0.08
2	< 1	1.00			1.00		
	1–2	1.20	1.04, 1.38		1.20	0.97, 1.49	
	3+	1.36	1.15, 1.61	0.001	1.33	1.08, 1.64	0.007
3	< 1	1.00			1.00		
	1–2	1.17	1.01, 1.35		1.17	0.94, 1.46	
	3+	1.30	1.10, 1.55	0.003	1.28	1.03, 1.58	0.03
4a	< 1	1.00			1.00		
	1–2	1.12	0.97, 1.30		1.13	0.91, 1.41	
	3+	1.14	0.97, 1.35	0.13	1.15	0.92, 1.44	0.25
Texting							
1	< 1	1.00			1.00		
	1–2	1.09	0.95, 1.25		1.01	0.87, 1.18	
	3+	1.22	0.96, 1.54	0.10	1.25	1.01, 1.56	0.06
2	< 1	1.00			1.00		
	1–2	0.98	0.83, 1.15		0.90	0.75, 1.09	
	3+	0.97	0.75, 1.25	0.78	1.00	0.78, 1.28	0.87
3	< 1	1.00			1.00		
	1–2	0.99	0.83, 1.19		0.93	0.77, 1.11	
	3+	1.00	0.78, 1.30	0.99	1.03	0.80, 1.33	0.91
4a	< 1	1.00			1.00		
	1–2	1.00	0.84, 1.20		0.95	0.79, 1.14	
	3+	1.00	0.77, 1.30	0.99	1.05	0.81, 1.35	0.81

Table displays models 1 to 3 and 4a in 100 multiply imputed datasets.

Model 1 was unadjusted.

Model 2 adjusted for sex, maternal age, anxiety at age 15, maternal anxiety and depression, maternal education, parental socioeconomic position.

Model 3 also adjusted for child IQ, parental conflict, presence of the child’s father, number of people living in the child’s home, bullying and family TV use in early life.

Model 4a further adjusted for time spent alone (weekdays or weekends, as applicable).

### Depression

Odds ratios for the association between watching television, computer use and texting and depression are shown in Table 3. After adjustment for potential confounders, there was no evidence for an association between time spent watching television or time spent texting and depression, on weekdays or weekends. Similarly, there was no clear evidence of an association between time spent using a computer on a weekday and depression (OR for 1–2 h = 1.04, 95% CI: 0.85 to 1.29; OR for 3+ hours = 1.13, 95% CI: 0.89 to 1.44, *p* for linear trend = 0.29, model 3; Table 3). Evidence of a small positive association was found between time spent using a computer on weekend days and depression (OR for 1–2 h = 1.12, 95% CI: 0.93 to 1.35; OR for 3+ hours = 1.35, 95% CI: 1.10 to 1.65 *p* for linear trend = 0.003, model 3). This association was only slightly attenuated by adjusting for time spent alone (OR for 1–2 h = 1.11, 95% CI: 0.92 to 1.35; OR for 3+ hours = 1.30, 95% CI: 1.06 to 1.58, *p* for linear trend = 0.007; Table 3). As was the case for anxiety, adjusting for other activities (apart from time spent alone) had very little impact on the odds ratios (Additional file 1, Tables S5-S7).

**Table 3:** Odds ratios for associations between depression and watching television, computer use and texting (n = 14,665).

		Week days			Weekend days		
Model	Hours of use	OR	95%CI	p-value	OR	95%CI	*p*-value
Television							
1	< 1	1.00			1.00		
	1–2	0.95	0.82, 1.11		0.91	0.75, 1.12	
	3+	1.12	0.92, 1.37	0.26	1.06	0.85, 1.31	0.44
2	< 1	1.00			1.00		
	1–2	0.99	0.83, 1.18		0.97	0.78, 1.20	
	3+	1.17	0.92, 1.49	0.19	1.13	0.89, 1.42	0.21
3	< 1	1.00			1.00		
	1–2	1.00	0.83, 1.20		0.97	0.79, 1.21	
	3+	1.16	0.91, 1.50	0.23	1.11	0.88, 1.41	0.27
4a	< 1	1.00			1.00		
	1–2	1.01	0.86, 1.17		0.97	0.78, 1.19	
	3+	1.14	0.86, 1.40	0.31	1.08	0.86, 1.36	0.40
Computer use							
1	< 1	1.00			1.00		
	1–2	1.01	0.82, 1.23		1.01	0.83, 1.23	
	3+	1.10	0.86, 1.41	0.40	1.21	0.99, 1.47	0.04
2	< 1	1.00			1.00		
	1–2	1.05	0.86, 1.30		1.15	0.95, 1.38	
	3+	1.18	0.93, 1.50	0.16	1.37	1.12, 1.68	0.001
3	< 1	1.00			1.00		
	1–2	1.04	0.85, 1.29		1.12	0.93, 1.35	
	3+	1.13	0.89, 1.44	0.29	1.35	1.10, 1.65	0.003
4a	< 1	1.00			1.00		
	1–2	1.03	0.83, 1.27		1.11	0.92, 1.35	
	3+	1.06	0.84, 1.35	0.60	1.30	1.06, 1.58	0.007
Texting							
1	< 1	1.00			1.00		
	1–2	1.15	1.01, 1.30		1.13	0.98, 1.30	
	3+	1.30	1.08, 1.57	0.002	1.29	1.09, 1.53	0.002
2	< 1	1.00			1.00		
	1–2	1.06	0.92, 1.23		1.03	0.87, 1.20	
	3+	1.02	0.84, 1.24	0.73	1.02	0.86, 1.23	0.76
3	< 1	1.00			1.00		
	1–2	1.05	0.90, 1.22		1.02	0.88, 1.20	
	3+	1.01	0.82, 1.23	0.86	1.02	0.85, 1.23	0.80
4a	< 1	1.00			1.00		
	1–2	1.05	0.91, 1.22		1.04	0.89, 1.21	
	3+	1.00	0.82, 1.23	0.89	1.02	0.85, 1.24	0.75

Table displays models 1 to 3 and 4a in 100 multiply imputed datasets.

Model 1 was unadjusted.

Model 2 adjusted for sex, depression at age 15 years, maternal age, maternal anxiety and depression, maternal education, parental socioeconomic position.

Model 3 also adjusted for child IQ, parental conflict, presence of the child’s father, number of people living in the child’s home, bullying and family TV use in early life.

Model 4a further adjusted for time spent alone (weekdays or weekends, as applicable).

### Sensitivity analyses

The results from the complete case analyses were generally consistent with those obtained using multiple imputation (although less precisely estimated), although the odds ratios for watching television on weekend days for both anxiety and depression were somewhat larger than those obtained using multiple imputation (Additional file 1, Tables S8 and S9). Nevertheless, the conclusions from these analyses were essentially the same. When all individuals with imputed anxiety/depression were re-categorised as one level higher than predicted in each imputed dataset (except when they were already predicted as being in the highest category), the association between computer use and both anxiety and depression were weaker (Additional file 1, Table S10). However, again the overall conclusion of no evidence for an association between watching television and texting and anxiety/depression but some evidence for a small association between computer use and both anxiety and depression remained the same.

## Discussion

Our results indicate that there is a small positive association between computer use at age 16 and both anxiety and depression two years later. Although the increase in the risk of developing anxiety and depression is small, given the high prevalence of screen use in young people, effects of small magnitude may still result in a substantial population burden and could therefore be clinically significant. Increased time spent alone attenuated the associations, particularly for anxiety.

The existing evidence regarding the association between screen use and anxiety is limited, whereas the evidence for an association between depression and screen time is more consistent [4, 10, 13]. However, these studies cannot tell us whether any associations are likely to be causal. Several studies have found evidence for an association between anxiety and screen time [5, 6], but none adjusted for time spent alone, which attenuated the association in our study. In addition, none of these studies were longitudinal, so it was not possible to establish the temporality of the association. Our findings for depression are in line with previous research suggesting there is an association with screen time when time spent alone is not adjusted for [12].

There are different possible explanations for the results relating to time spent alone. It is possible that the measure of time alone used in this study may be measuring variance in anxiety or depression rather than confounding the relationship. Alternatively, time alone and screen time could be common markers of underlying causes of depression such as family circumstances or peer relationships. Our research highlights that time spent alone is an important factor (potentially as a confounder or as a marker of depression or anxiety) in this association that until now has been overlooked.

Besides time spent alone, various other mechanisms could explain the associations found between screen time and both anxiety and depression. Screen time allows for social comparisons with both fictional characters and real people who are perceivably higher up the social ladder than the viewer. In support of this theory, negative social comparisons on social networking sites are related to higher levels of depression and anxiety [29]. Cyber bullying (whereby individuals are bullied via social media and texting) could also partly explain this association; victims report feeling depressed and worried as a consequence of cyber bullying [30]. Alternatively, the sedentary nature of the screen time measured in this study may be the mechanism by which screen time and anxiety and depression are associated, as sedentary behaviour has been shown to be associated with both [4, 31].

A common criticism in the reviews of the literature is the widespread use of cross-sectional rather than longitudinal data [10]. An important strength of our study was the use of data from a longitudinal study and, in particular, our ability to adjust for previously identified anxiety and depression. Another strength of our study was the ability to adjust for a wide range of potential confounders. Nonetheless, there may be important confounders that were not measured in ALSPAC, or that were measured imperfectly. As a result, there is potential for residual confounding.

Another limitation is the extent of missing data; the proportion of individuals with complete data was low, which could have resulted in bias. We found evidence that individuals with anxiety and depression at age 7 years were more likely to have missing anxiety and depression data at age 18. This suggests (but cannot establish for certain) that the outcome data – depression and anxiety – were MNAR conditional on the covariates included in the analysis model – that is, the probability that depression and anxiety data were missing depended on their (unknown) missing values, even after taking account of the observed variables. If an outcome measure is MNAR then both a complete case analysis and MI will generally produce biased estimates of exposure-outcome associations, although there are exceptions to this if the outcome is binary [32]. However, since we had four earlier measures of anxiety and depression (that were more complete than the measures at 18 years), we were able to include these as auxiliary variables in the MI models, thus giving a better approximation to MAR and hence reducing the likelihood of bias [33]. The results for depression were generally weaker in the MI models, indicating some bias may have been present in the complete case analysis, although we cannot determine whether we have eliminated bias by using MI. We carried out sensitivity analyses making the assumption that imputed values of anxiety and depression were underestimates; although this weakened the results, the general conclusions remained the same.

A final limitation of our study is that screen use patterns have changed over time [34], and the data for the current study were gathered between 2007 and 2009. This predates the wide availability of smart phones, smart watches and tablets that allow for use of screens (and particularly social media which was not assessed in this study) at times and in situations where screen use may have previously been limited. It is difficult to ascertain whether the findings of this study would apply to young people and screen use today, and evidence actually suggests that increased screen time using more recent technology may have positive effects on social capital [35]. Additionally, screen time no longer necessarily means sedentary behaviour - some screen-based games, such as Pokemon Go, even encourage physical activity [36]. There is clearly a need to capture different aspects of screen time including the context and amount of time spent using screen-based devices and types of devices as well as types and range of different activities being undertaken in order to fully investigate how screen time affects mental health in young people. Recent research by Przybylski and Weinstein [37] suggested that moderate screen use may be beneficial. In their study, between one and four hours (depending on the type of activity) was found to be beneficial but was negatively associated with wellbeing above this threshold. They also found that any beneficial effects depended on whether use was on weekdays or weekends with negative effects on wellbeing seen at lower thresholds of use on weekdays. We found some differences between the effects of weekday and weekend exposure. However, the highest category of screen time measured in our study was three or more hours; as such, we could not differentiate between moderately high and very high use – and, as a consequence, could not assess whether there was a stronger association with very high levels of screen time. Furthermore, the categorisation of the screen time measure used in this analysis may not have been sensitive enough to detect moderate use between 2 and 3 h. As is the nature of secondary data, we were unable to create a category for 2 to 3 h due to the wording of the answer options provided in the questionnaire.

Different types of screen time may have different effects, both in terms of wellbeing and in terms of poor mental health. In their study, Przybylski and Weinstein found that different types of screen use had different effects on wellbeing [37]. For example, there was a negative linear trend for smart phone use on weekends in relation to wellbeing whereas there were positive trends for TV, computer or video game use below the pivot point for beneficial vs non-beneficial use. We also found differences between type of screen use, where only time spent on the computer was clearly associated with an increase in anxiety and depression whereas there was little evidence of associations with time spent texting or watching television. Evidently, the association between screen use and mental health is complex and there is not a linear association between simply any type of screen use and mental health. This difference could be due to the use of social networking sites, which were primarily accessed through computers at the time of the study, whereby negative social comparisons may be the mechanism of the association found. Alternatively, texting could be associated with social behaviour whereas computer use could be associated with exam and work-related stress. Another theory to explain the difference is that some screen types may induce effects at lower levels of exposure than others, perhaps due to perceived level of immersion; young people may be more likely to multi-task when watching TV, and texting is intermittent whereas computer use may be more focussed and continuous. The pattern of association between computer use and anxiety also differs from the association between computer use and depression. Where the effects for anxiety seem to be consistent across the time of the week, the association for depression is much stronger with computer use on weekends than weekdays suggesting the mechanisms underlying these effects may be different for anxiety and depression. This highlights the need for on-going research in the area to assess the effect of specific types of activity on mental health in young adults in order to provide up-to-date, accurate advice for screen use.

## Conclusions

In summary, our results suggest that increased computer use at age 16 is associated with an increased risk of depression and anxiety at age 18, although causality cannot be ascertained. After adjustment for potential confounders, there was little evidence of an effect of time spent texting or watching TV on risk of anxiety and depression indicating there may be a more complex relationship between screen time and mental health outcomes than simply more screen time increasing risk. Additionally, the size and strength of the associations differ depending on the time of the week the devices are used, suggesting further complexities in the relationship. Further research is needed to capture a wider range of use to distinguish between moderate through to very high screen time, and with more up-to-date screen time.

## Additional files

Additional file 1: Supplementary material. (DOCX 96 kb)

## Abbreviations

ALSPAC: Avon Longitudinal Study of Parents and Children
CI: Confidence interval
CIS-R: Revised Computerised Interview Schedule
CSE: Certificate of Secondary Education
DAWBA: Development and Well-Being Assessment
EPDS: Edinburgh Post-Natal Depression Scale
ICD-10: International Statistical Classification of Diseases and Related Health Problems 10th Revision
MAR: Missing at random
MI: Multiple imputation
MNAR: Missing not at random
OR: Odds ratio
SES: Socioeconomic Status
WISC-III^UK^: Wechsler Intelligence Scale for children
