# Supplementary material for: Is screen time associated with anxiety or depression in young people? Results from a UK birth cohort
Source: BMC Public Health. 2019 Jan 17;19:82. doi: 10.1186/s12889-018-6321-9 (PMC6337855; doi:10.1186/s12889-018-6321-9)
Supplement: Supplementary file 1 — Supplementary material. (DOCX 96 kb) [file 12889_2018_6321_MOESM1_ESM.docx]

**Supplementary Material**

1. **Additional information regarding study variables**

Further information is provided here regarding variables which were not gathered from standardised questionnaires. Some of these variables were derived from a number of questions; all of these questions and the methods for deriving the variables are shown below. Other additional information regarding the study variables such as definitions of maternal education have also been included.

**Screen time and other activity variables**

Screen time and time spent engaging in other activities on weekdays and weekend days were determined using the following questions:

**How much time on average do you spend each day?**

|  | 1. On a typical weekday | | |  | | | 1. On a typical weekend day | | |
| --- | --- | --- | --- | --- | --- | --- | --- | --- | --- |
| 1. In a car, bus or other transport | 0) Not at all | 1) Less than one hour | 2) 1-2 hours | 3) 3 or more hours |  | 0) Not at all | 1) Less than one hour | 2) 1-2 hours | 3) 3 or more hours |
| 1. Out of doors in summer | 0) Not at all | 1) Less than one hour | 2) 1-2 hours | 3) 3 or more hours |  | 0) Not at all | 1) Less than one hour | 2) 1-2 hours | 3) 3 or more hours |
| 1. Out of doors in winter | 0) Not at all | 1) Less than one hour | 2) 1-2 hours | 3) 3 or more hours |  | 0) Not at all | 1) Less than one hour | 2) 1-2 hours | 3) 3 or more hours |
| 1. Watching TV | 0) Not at all | 1) Less than one hour | 2) 1-2 hours | 3) 3 or more hours |  | 0) Not at all | 1) Less than one hour | 2) 1-2 hours | 3) 3 or more hours |
| 1. With other young people | 0) Not at all | 1) Less than one hour | 2) 1-2 hours | 3) 3 or more hours |  | 0) Not at all | 1) Less than one hour | 2) 1-2 hours | 3) 3 or more hours |
| 1. Drawing, making, constructing things | 0) Not at all | 1) Less than one hour | 2) 1-2 hours | 3) 3 or more hours |  | 0) Not at all | 1) Less than one hour | 2) 1-2 hours | 3) 3 or more hours |
| 1. Doing things by yourself | 0) Not at all | 1) Less than one hour | 2) 1-2 hours | 3) 3 or more hours |  | 0) Not at all | 1) Less than one hour | 2) 1-2 hours | 3) 3 or more hours |
| 1. School or college homework | 0) Not at all | 1) Less than one hour | 2) 1-2 hours | 3) 3 or more hours |  | 0) Not at all | 1) Less than one hour | 2) 1-2 hours | 3) 3 or more hours |
| 1. Reading books for pleasure | 0) Not at all | 1) Less than one hour | 2) 1-2 hours | 3) 3 or more hours |  | 0) Not at all | 1) Less than one hour | 2) 1-2 hours | 3) 3 or more hours |
| 1. Playing musical instruments | 0) Not at all | 1) Less than one hour | 2) 1-2 hours | 3) 3 or more hours |  | 0) Not at all | 1) Less than one hour | 2) 1-2 hours | 3) 3 or more hours |
| 1. Using a computer | 0) Not at all | 1) Less than one hour | 2) 1-2 hours | 3) 3 or more hours |  | 0) Not at all | 1) Less than one hour | 2) 1-2 hours | 3) 3 or more hours |
| 1. Talking on a mobile phone | 0) Not at all | 1) Less than one hour | 2) 1-2 hours | 3) 3 or more hours |  | 0) Not at all | 1) Less than one hour | 2) 1-2 hours | 3) 3 or more hours |
| 1. Texting | 0) Not at all | 1) Less than one hour | 2) 1-2 hours | 3) 3 or more hours |  | 0) Not at all | 1) Less than one hour | 2) 1-2 hours | 3) 3 or more hours |
| 1. Talking on an ordinary phone | 0) Not at all | 1) Less than one hour | 2) 1-2 hours | 3) 3 or more hours |  | 0) Not at all | 1) Less than one hour | 2) 1-2 hours | 3) 3 or more hours |

For the measure of exercise, participants were asked how often they had exercised in a typical week during the past year; possible responses were:

0. Never 1. Less than once a month 2. 1-3 times per month 3. 1-4 times per week 4. 5 or more times a week.

For all these other activities, categories were combined where there were small numbers.

**Maternal education**

Maternal education was defined as the mother’s highest educational qualification in a 4-level categorical variable (CSE/vocational/lower; O level; A level; degree/higher). CSEs (Certificate of Secondary Education) and O levels were qualifications taken at age 16 – now replaced by GCSEs (General Certificate of Secondary Education) in England, Wales and Northern Ireland. A levels are exams taken at age 18 in these countries.

**Socioeconomic status**

Socio-economic status was based on the higher of the mother or partner's occupational social class using the 1991 British Office of Population and Census Statistics classification.

**Parental conflict**

Parental conflict was determined using two questions relating to physical and emotional cruelty (see below) and a partner aggression score derived from an aggression affection questionnaire when the child was 8 months old. If participants gave response one to either emotional or physical cruelty question below or if when summed their aggression score was 6 or below, they were categorised as having partner conflict.

Emotional and physical cruelty questions:

Have any of these occurred since the baby was born? If so, please assess how much effect it had on you.

a) Your partner was physically cruel.

1. Yes & affected me a lot 2. Yes, moderately affected 3. Yes, mildly affected

4. Yes, but did not affect me at all 5. No did not happen

b) Your partner was emotionally cruel.

1. Yes & affected me a lot 2. Yes, moderately affected 3. Yes, mildly affected

4. Yes, but did not affect me at all 5. No did not happen

Aggression score questions:

How frequently does each description fit your own partnership?

Does your partner get angry with you?

1. Very often 2. Often 3. Sometimes 4. Rarely 5. Never

Do you have arguments with your partner?

1. Very often 2. Often 3. Sometimes 4. Rarely 5. Never

Do you get angry with your partner?

1. Very often 2. Often 3. Sometimes 4. Rarely 5. Never

**Presence of the father in the home**

Presence of the father in the home was determined by one question:

Does the biological (natural) father of the study child live with the study child?

1. Yes 1. No

**Number of people living in the home**

Total number of people living in the household was determined by one question:

How many people live in your household now? (including yourself)

**Bullying status**

One question was used to determine whether a child had been bullied after the age of 12:

Did this happen to you since you were aged 12... Bullying by another person?

1. Yes 0. No

**Early family TV use**

Early family TV use was determined by three questions and the answers summed to create a score:

Do you usually have the television on...

1. In the mornings?
2. In the afternoons?
3. In the evenings?

3. Yes, every day 2. Yes, some days 1. No, hardly ever

1. **Multiple imputation procedure**

All participants included in the analysis (14,665) had complete data on sex and maternal age at birth. Tabe S1 details each variable included in the imputation models. For each set of imputations 100 datasets were imputed; Stata’s *mi impute* command was used.

**Table S1: Variables used in the multiple imputation models (n=14,665)**

| **Variable** | Type of variable (number of categories if categorical) | Number (%) with missing data | Regression model used to predict missing data in this variable |
| --- | --- | --- | --- |
| **Outcome variables** |  |  |  |
| Anxiety | Categorical (3) | 10,103 (69%) | Multinomial logistic |
| Depression | Categorical (3) | 10,103 (69%) | Multinomial logistic |
| Exposure variables |  |  |  |
| TV use, weekdays | Categorical (3)^1^ | 9,593 (65%) | Multinomial logistic |
| TV use, weekends | Categorical (3)^1^ | 9,765 (67%) | Multinomial logistic |
| Computer use, weekdays | Categorical (3)^1^ | 9,597 (65%) | Multinomial logistic |
| Computer use, weekends | Categorical (3)^1^ | 9,764 (67%) | Multinomial logistic |
| Texting, weekdays | Categorical (3)^1^ | 9,600 (66%) | Multinomial logistic |
| Texting, weekends | Categorical (3)^1^ | 9,764 (67%) | Multinomial logistic |
| Covariates |  |  |  |
| Sex | Binary | 0 | N/A |
| Maternal age | Categorical (3) | 711 (5%) | Multinomial logistic |
| Maternal education | Categorical (4) | 2,270 (16%) | Multinomial logistic |
| Family socioeconomic position | Binary | 3,180 (22%) | Logistic |
| Parental conflict | Binary | 3,352 (23%) | Logistic |
| Number living in home | Discrete numerical | 5,193 (35%) | Truncated linear regression |
| Presence of father | Binary | 5,178 (35%) | Logistic |
| Child been bullied | Binary | 9,605 (66%) | Logistic |
| Maternal anxiety | Binary | 5,273 (36%) | Logistic |
| Maternal depression | Discrete numerical | 3,492 (24%) | Linear regression |
| Family TV score | Discrete numerical | 4,011 (27%) | Truncated linear regression |
| Child IQ | Discrete numerical | 7,324 (50%) | Linear regression |
| Time spent exercising | Categorical (3) | 9,629 (66%) | Multinomial logistic |
| Time spent on public transport, weekdays | Binary | 9,590 (65%) | Logistic |
| Time spent on public transport, weekends | Binary | 9,751 (66%) | Logistic |
| Time spent outdoors in summer, weekdays | Binary | 9,602 (65%) | Logistic |
| Time spent outdoors in summer, weekends | Binary | 9,762 (66%) | Logistic |
| Time spent outdoors in winter, weekdays | Categorical (3) | 9,603 (65%) | Multinomial logistic |
| Time spent outdoors in winter, weekends | Categorical (3) | 9,771 (67%) | Multinomial logistic |
| Time spent playing with others, weekdays | Binary | 9,596 (65%) | Logistic |
| Time spent playing with others, weekends | Binary | 9,765 (66%) | Logistic |
| Time spent drawing, making, weekdays | Categorical (3) | 9,601 (65%) | Multinomial logistic |
| Time spent drawing, making, weekends | Categorical (3) | 9,763 (66%) | Multinomial logistic |
| Time spent alone, weekdays | Categorical (3) | 9,611 (66%) | Multinomial logistic |
| Time spent alone, weekends | Categorical (3) | 9,774 (67%) | Multinomial logistic |
| Time spent doing school work, weekdays | Categorical (3) | 9,604 (65%) | Multinomial logistic |
| Time spent doing school work, weekends | Categorical (3) | 9,770 (67%) | Multinomial logistic |
| Time spent reading, weekdays | Categorical (3) | 9,609 (66%) | Multinomial logistic |
| Time spent reading, weekends | Categorical (3) | 9,763 (67%) | Multinomial logistic |
| Time spent playing musical instruments, weekdays | Binary | 9,603 (65%) | Logistic |
| Time spent playing musical instruments, weekends | Binary | 9,767 (67%) | Logistic |
| Time spent talking on a mobile, weekdays | Categorical (3) | 9,600 (65%) | Multinomial logistic |
| Time spent talking on a mobile, weekends | Categorical (3) | 9,762 (67%) | Multinomial logistic |
| Time spent talking on a landline, weekdays | Binary | 9,603 (65%) | Logistic |
| Time spent talking on a landline, weekends | Binary | 9,754 (67%) | Logistic |
| Auxiliary variables (i.e. variables not included in the analysis model) |  |  |  |
| Paternal education | Categorical (4) | 3,962 (27%) | Multinomial logistic |
| Parity | Categorical (5) | 1,757 (12%) | Multinomial logistic |
| Anxiety (DAWBA) at 7 years, parent-reported | Binary | 6430 (44%) | Logistic |
| Anxiety (DAWBA) at 10 years, parent-reported | Binary | 6852 (47%) | Logistic |
| Anxiety (DAWBA) at 13 years, parent-reported | Binary | 7,566 (52%) | Logistic |
| Anxiety (DAWBA at 15 years, child-reported | Categorical (3) | 9,303 (63%) | Multinomial logistic |
| Depression (DAWBA) at 7 years, parent-reported | Binary | 6,592 (45%) | Logistic |
| Depression (DAWBA) at 10 years, parent-reported | Binary | 7,018 (48%) | Logistic |
| Depression (DAWBA) at 13 years, parent-reported | Binary | 7,714 (53%) | Logistic |
| Depression (DAWBA at 15 years, child-reported | Categorical (3) | 9,305 (63%) | Multinomial logistic |
| Weekday (landline) phone use (14 years) | Binary | 8,536 (58%) | Logistic |
| Weekend (landline) phone use (14 years) | Binary | 8.664 (59%) | Logistic |
| Weekday mobile use (14 years) | Categorical (3) | 8,535 (58%) | Multinomial logistic |
| Weekend mobile use (14 years) | Categorical (3) | 8,664 (59%) | Multinomial logistic |
| Presence of father (8 years) | Binary | 6,797 (46%) | Logistic |
| Presence of father (10 years) | Binary | 6,573 (45%) | Logistic |
| Child does creative activities (14 years) | Binary | 7,645 (52%) | Logistic |
| Child plays musical instrument (13 years) | Binary | 7,727 (53%) | Logistic |
| Child has TV in bedroom (9 years) | Binary | 6,360 (43%) | Logistic |
| Child has TV in bedroom (16 years) | Binary | 8,951 (61%) | Logistic |
| Child has own mobile phone (10 years) | Binary | 6,364 (43%) | Logistic |

**Table S2. Odds ratios for associations between anxiety and watching television for** **models 4b-4l (100 multiply imputed datasets; n=14,665)**

|  | | Week days | | | Weekend days | | |
| --- | --- | --- | --- | --- | --- | --- | --- |
| **Model** | Hours of use | OR | 95%CI | p-value | OR | 95%CI | p-value |
| 4b | <1  1-2  3+ | 1.00  1.02  1.11 | 0.87, 1.19  0.91, 1.36 | 0.29 | 1.00  1.07  1.09 | 0.89, 1.28  0.89, 1.33 | 0.43 |
| 4c | <1  1-2  3+ | 1.00  1.02  1.12 | 0.87, 1.19  0.92, 1.37 | 0.27 | 1.00  1.07  1.10 | 0.89, 1.28  0.91, 1.33 | 0.34 |
| 4d | <1  1-2  3+ | 1.00  1.03  1.13 | 0.88, 1.20  0.92, 1.38 | 0.25 | 1.00  1.07  1.08 | 0.89, 1.28  0.90, 1.31 | 0.42 |
| 4e | <1  1-2  3+ | 1.00  1.02  1.13 | 0.87, 1.20  0.92, 1.38 | 0.25 | 1.00  1.06  1.08 | 0.89, 1.27  0.89, 1.31 | 0.45 |
| 4f | <1  1-2  3+ | 1.00  1.02  1.12 | 0.87, 1.19  0.91, 1.37 | 0.27 | 1.00  1.06  1.08 | 0.89, 1.27  0.89, 1.31 | 0.43 |
| 4g | <1  1-2  3+ | 1.00  1.02  1.11 | 0.87, 1.19  0.91, 1.36 | 0.29 | 1.00  1.07  1.09 | 0.89, 1.28  0.90, 1.32 | 0.42 |
| 4h | <1  1-2  3+ | 1.00  1.02  1.12 | 0.87, 1.20  0.92, 1.37 | 0.27 | 1.00  1.05  1.06 | 0.88, 1.25  0.88, 1.28 | 0.56 |
| 4i | <1  1-2  3+ | 1.00  1.02  1.12 | 0.87, 1.20  0.91, 1.37 | 0.27 | 1.00  1.04  1.07 | 0.87, 1.25  0.88, 1.30 | 0.50 |
| 4j | <1  1-2  3+ | 1.00  1.03  1.15 | 0.88, 1.21  0.93, 1.41 | 0.19 | 1.00  1.07  1.11 | 0.90, 1.28  0.92, 1.34 | 0.28 |
| 4k | <1  1-2  3+ | 1.00  1.02  1.12 | 0.87, 1.19  0.92, 1.37 | 0.26 | 1.00  1.06  1.09 | 0.89, 1.27  0.90, 1.32 | 0.41 |
| 4l | <1  1-2  3+ | 1.00  1.02  1.11 | 0.87, 1.19  0.91, 1.36 | 0.29 | 1.00  1.06  1.08 | 0.89, 1.27  0.90, 1.31 | 0.42 |
| Models 4b-4l adjusted for sex, previous anxiety (at age 15 years), maternal age, maternal anxiety and depression, maternal education, parental socioeconomic position, child IQ, parental conflict, presence of the child’s father, number of people living in the child’s home, bullying and family television use in early life. Each model was further adjusted for time spent engaging in one other activity on weekdays and weekends (time spent exercising [model 4b], time spent in a car/bus/train [model 4c], time spent outdoors in summer [model 4d], time spent playing outdoors in winter [model 4e], time spent playing with others [model 4f], time spent drawing, making or constructing things [model 4g], time spent on school or college work [model 4h], time spent reading [model 4i], time spent playing musical instruments [model 4j], time spent talking on a mobile phone [model 4k] and time spent talking on a landline phone [model 4l]. | | | | | | | |

**Table S3. Odds ratios for associations between anxiety and computer use for models 4b-4l (100 multiply imputed datasets; n=14,665)**

|  | | Week days | | | Weekend days | | |
| --- | --- | --- | --- | --- | --- | --- | --- |
| **Model** | Hours of use | OR | 95%CI | p-value | OR | 95%CI | p-value |
| 4b | <1  1-2  3+ | 1.00  1.16  1.30 | 1.01, 1.35  1.09, 1.54 | 0.004 | 1.00  1.18  1.27 | 0.94, 1.47  1.03, 1.57 | 0.03 |
| 4c | <1  1-2  3+ | 1.00  1.17  1.31 | 1.01, 1.35  1.10, 1.55 | 0.003 | 1.00  1.18  1.28 | 0.95, 1.47  1.04, 1.60 | 0.02 |
| 4d | <1  1-2  3+ | 1.00  1.17  1.32 | 1.01, 1.36  1.11, 1.57 | 0.002 | 1.00  1.17  1.27 | 0.94, 1.46  1.02, 1.57 | 0.03 |
| 4e | <1  1-2  3+ | 1.00  1.17  1.32 | 1.01, 1.35  1.11, 1.57 | 0.002 | 1.00  1.18  1.27 | 0.95, 1.47  1.02, 1.57 | 0.03 |
| 4f | <1  1-2  3+ | 1.00  1.16  1.30 | 1.00, 1.35  1.09, 1.55 | 0.004 | 1.00  1.17  1.27 | 0.94, 1.46  1.02, 1.58 | 0.03 |
| 4g | <1  1-2  3+ | 1.00  1.16  1.30 | 1.00, 1.34  1.09, 1.54 | 0.004 | 1.00  1.17  1.27 | 0.94, 1.45  1.02, 1.57 | 0.03 |
| 4h | <1  1-2  3+ | 1.00  1.17  1.29 | 1.01, 1.36  1.08, 1.55 | 0.006 | 1.00  1.15  1.24 | 0.92, 1.43  0.99, 1.55 | 0.06 |
| 4i | <1  1-2  3+ | 1.00  1.15  1.29 | 1.00, 1.33  1.09, 1.53 | 0.004 | 1.00  1.16  1.25 | 0.93, 1.43  1.01, 1.55 | 0.03 |
| 4j | <1  1-2  3+ | 1.00  1.16  1.30 | 1.00, 1.35  1.09, 1.54 | 0.004 | 1.00  1.17  1.27 | 0.94, 1.45  1.02, 1.58 | 0.03 |
| 4k | <1  1-2  3+ | 1.00  1.18  1.33 | 1.02, 1.36  1.12, 1.58 | 0.001 | 1.00  1.18  1.29 | 0.95, 1.46  1.04, 1.60 | 0.02 |
| 4l | <1  1-2  3+ | 1.00  1.16  1.29 | 1.00, 1.34  1.09, 1.54 | 0.004 | 1.00  1.17  1.27 | 0.94, 1.45  1.03, 1.57 | 0.03 |
| Models 4b-4l adjusted for sex, previous anxiety (at age 15 years), maternal age, maternal anxiety and depression, maternal education, parental socioeconomic position, child IQ, parental conflict, presence of the child’s father, number of people living in the child’s home, bullying and family television use in early life. Each model was further adjusted for time spent engaging in one other activity on weekdays and weekends (time spent exercising [model 4b], time spent in a car/bus/train [model 4c], time spent outdoors in summer [model 4d], time spent playing outdoors in winter [model 4e], time spent playing with others [model 4f], time spent drawing, making or constructing things [model 4g], time spent on school or college work [model 4h], time spent reading [model 4i], time spent playing musical instruments [model 4j], time spent talking on a mobile phone [model 4k] and time spent talking on a landline phone [model 4l]. | | | | | | | |

**Table S4. Odds ratios for associations between anxiety and texting for models 4b-4l (100 multiply imputed datasets; n=14,665)**

|  | | Week days | | | Weekend days | | |
| --- | --- | --- | --- | --- | --- | --- | --- |
| **Model** | Hours of use | OR | 95%CI | p-value | OR | 95%CI | p-value |
| 4b | <1  1-2  3+ | 1.00  0.99  1.00 | 0.83, 1.18  0.78, 1.30 | 0.99 | 1.00  0.92  1.03 | 0.76, 1.11  0.80, 1.31 | 0.92 |
| 4c | <1  1-2  3+ | 1.00  1.00  1.01 | 0.84, 1.19  0.78, 1.30 | 0.96 | 1.00  0.94  1.05 | 0.78, 1.13  0.81, 1.35 | 0.80 |
| 4d | <1  1-2  3+ | 1.00  1.02  1.05 | 0.86, 1.22  0.81, 1.36 | 0.69 | 1.00  0.94  1.06 | 0.78, 1.13  0.82, 1.36 | 0.75 |
| 4e | <1  1-2  3+ | 1.00  1.01  1.03 | 0.84, 1.20  0.79, 1.34 | 0.83 | 1.00  0.94  1.05 | 0.78, 1.13  0.81, 1.36 | 0.80 |
| 4f | <1  1-2  3+ | 1.00  0.99  1.00 | 0.83, 1.18  0.78, 1.29 | 0.99 | 1.00  0.93  1.05 | 0.77, 1.13  0.81, 1.37 | 0.79 |
| 4g | <1  1-2  3+ | 1.00  0.99  1.00 | 0.83, 1.19  0.77, 1.30 | 0.99 | 1.00  0.93  1.05 | 0.77, 1.12  0.81, 1.35 | 0.84 |
| 4h | <1  1-2  3+ | 1.00  0.99  1.00 | 0.83, 1.18  0.77, 1.29 | 0.96 | 1.00  0.93  1.05 | 0.77, 1.12  0.81, 1.36 | 0.82 |
| 4i | <1  1-2  3+ | 1.00  0.99  1.03 | 0.83, 1.19  0.80, 1.33 | 0.85 | 1.00  0.94  1.06 | 0.78, 1.13  0.83, 1.37 | 0.73 |
| 4j | <1  1-2  3+ | 1.00  1.00  1.02 | 0.84, 1.19  0.79, 1.32 | 0.90 | 1.00  0.94  1.05 | 0.78, 1.13  0.82, 1.36 | 0.79 |
| 4k | <1  1-2  3+ | 1.00  1.03  1.08 | 0.86, 1.23  0.84, 1.39 | 0.55 | 1.00  0.97  1.13 | 0.79, 1.20  0.82, 1.54 | 0.50 |
| 4l | <1  1-2  3+ | 1.00  0.99  1.00 | 0.83, 1.18  0.77, 1.29 | 0.98 | 1.00  0.92  1.03 | 0.76, 1.11  0.80, 1.33 | 0.93 |
| Models 4b-4l adjusted for sex, previous anxiety (at age 15 years), maternal age, maternal anxiety and depression, maternal education, parental socioeconomic position, child IQ, parental conflict, presence of the child’s father, number of people living in the child’s home, bullying and family television use in early life. Each model was further adjusted for time spent engaging in one other activity on weekdays and weekends (time spent exercising [model 4b], time spent in a car/bus/train [model 4c], time spent outdoors in summer [model 4d], time spent playing outdoors in winter [model 4e], time spent playing with others [model 4f], time spent drawing, making or constructing things [model 4g], time spent on school or college work [model 4h], time spent reading [model 4i], time spent playing musical instruments [model 4j], time spent talking on a mobile phone [model 4k] and time spent talking on a landline phone [model 4l]. | | | | | | | |

**Table S5. Odds ratios for associations between depression and watching television for models 4b-4l (100 multiply imputed datasets; n=14,665)**

|  | | Week days | | | Weekend days | | |
| --- | --- | --- | --- | --- | --- | --- | --- |
| **Model** | Hours of use | OR | 95%CI | p-value | OR | 95%CI | p-value |
| 4b | <1  1-2  3+ | 1.00  0.99  1.14 | 0.82, 1.19  0.88, 1.46 | 0.29 | 1.00  0.98  1.10 | 0.79, 1.21  0.87, 1.40 | 0.31 |
| 4c | <1  1-2  3+ | 1.00  1.00  1.17 | 0.83, 1.21  0.91, 1.50 | 0.23 | 1.00  0.97  1.12 | 0.79, 1.21  0.88, 1.42 | 0.27 |
| 4d | <1  1-2  3+ | 1.00  1.00  1.17 | 0.83, 1.21  0.91, 1.50 | 0.23 | 1.00  0.98  1.11 | 0.79, 1.21  0.88, 1.40 | 0.28 |
| 4e | <1  1-2  3+ | 1.00  1.00  1.17 | 0.83, 1.21  0.91, 1.50 | 0.23 | 1.00  0.97  1.11 | 0.78, 1.21  0.88, 1.40 | 0.30 |
| 4f | <1  1-2  3+ | 1.00  1.00  1.16 | 0.83, 1.20  0.91, 1.50 | 0.23 | 1.00  0.97  1.11 | 0.78, 1.20  0.87, 1.40 | 0.31 |
| 4g | <1  1-2  3+ | 1.00  1.00  1.16 | 0.83, 1.20  0.90, 1.49 | 0.25 | 1.00  0.97  1.11 | 0.79, 1.21  0.88, 1.41 | 0.29 |
| 4h | <1  1-2  3+ | 1.00  1.01  1.17 | 0.83, 1.22  0.91, 1.50 | 0.23 | 1.00  0.97  1.11 | 0.79, 1.20  0.88, 1.40 | 0.29 |
| 4i | <1  1-2  3+ | 1.00  1.00  1.17 | 0.83, 1.21  0.90, 1.50 | 0.24 | 1.00  0.96  1.10 | 0.77, 1.19  0.87, 1.39 | 0.32 |
| 4j | <1  1-2  3+ | 1.00  1.01  1.18 | 0.84, 1.21  0.92, 1.52 | 0.18 | 1.00  0.98  1.13 | 0.79, 1.21  0.90, 1.43 | 0.21 |
| 4k | <1  1-2  3+ | 1.00  1.00  1.17 | 0.83, 1.21  0.91, 1.50 | 0.23 | 1.00  0.97  1.11 | 0.79, 1.20  0.88, 1.41 | 0.28 |
| 4l | <1  1-2  3+ | 1.00  1.00  1.16 | 0.83, 1.20  0.90, 1.50 | 0.24 | 1.00  0.97  1.11 | 0.78, 1.21  0.88, 1.41 | 0.29 |
| Models 4b-4l adjusted for sex, previous depression (at age 15 years), maternal age, maternal anxiety and depression, maternal education, parental socioeconomic position, child IQ, parental conflict, presence of the child’s father, number of people living in the child’s home, bullying and family television use in early life. Each model was further adjusted for time spent engaging in one other activity on weekdays and weekends (time spent exercising [model 4b], time spent in a car/bus/train [model 4c], time spent outdoors in summer [model 4d], time spent playing outdoors in winter [model 4e], time spent playing with others [model 4f], time spent drawing, making or constructing things [model 4g], time spent on school or college work [model 4h], time spent reading [model 4i], time spent playing musical instruments [model 4j], time spent talking on a mobile phone [model 4k] and time spent talking on a landline phone [model 4l]. | | | | | | | |

**Table S6. Odds ratios for associations between depression and computer use for models 4b-4l (100 multiply imputed datasets; n=14,665)**

|  | | Week days | | | Weekend days | | |
| --- | --- | --- | --- | --- | --- | --- | --- |
| **Model** | Hours of use | OR | 95%CI | p-value | OR | 95%CI | p-value |
| 4b | <1  1-2  3+ | 1.00  1.05  1.12 | 0.85, 1.29  0.88, 1.42 | 0.36 | 1.00  1.12  1.34 | 0.92, 1.36  1.08, 1.64 | 0.005 |
| 4c | <1  1-2  3+ | 1.00  1.05  1.14 | 0.85, 1.29  0.90, 1.44 | 0.26 | 1.00  1.12  1.35 | 0.93, 1.36  1.10, 1.65 | 0.003 |
| 4d | <1  1-2  3+ | 1.00  1.05  1.14 | 0.85, 1.29  0.89, 1.45 | 0.28 | 1.00  1.12  1.34 | 0.93, 1.35  1.10, 1.63 | 0.003 |
| 4e | <1  1-2  3+ | 1.00  1.05  1.13 | 0.85, 1.29  0.89, 1.44 | 0.29 | 1.00  1.12  1.34 | 0.93, 1.36  1.09, 1.64 | 0.004 |
| 4f | <1  1-2  3+ | 1.00  1.04  1.13 | 0.84, 1.29  0.89, 1.44 | 0.30 | 1.00  1.12  1.34 | 0.93, 1.35  1.09, 1.64 | 0.004 |
| 4g | <1  1-2  3+ | 1.00  1.04  1.12 | 0.84, 1.28  0.88, 1.43 | 0.32 | 1.00  1.12  1.34 | 0.93, 1.35  1.09, 1.64 | 0.004 |
| 4h | <1  1-2  3+ | 1.00  1.06  1.14 | 0.86, 1.31  0.89, 1.44 | 0.29 | 1.00  1.12  1.34 | 0.93, 1.36  1.09, 1.64 | 0.004 |
| 4i | <1  1-2  3+ | 1.00  1.04  1.13 | 0.84, 1.28  0.89, 1.44 | 0.29 | 1.00  1.11  1.33 | 0.91, 1.34  1.08, 1.64 | 0.005 |
| 4j | <1  1-2  3+ | 1.00  1.03  1.13 | 0.84, 1.29  0.89, 1.44 | 0.30 | 1.00  1.12  1.34 | 0.92, 1.35  1.09, 1.64 | 0.004 |
| 4k | <1  1-2  3+ | 1.00  1.04  1.14 | 0.85, 1.29  0.89, 1.45 | 0.27 | 1.00  1.12  1.35 | 0.93, 1.36  1.10, 1.66 | 0.003 |
| 4l | <1  1-2  3+ | 1.00  1.04  1.13 | 0.85, 1.28  0.89, 1.43 | 0.29 | 1.00  1.12  1.34 | 0.92, 1.35  1.10, 1.64 | 0.003 |
| Models 4b-4l adjusted for sex, previous depression (at age 15 years), maternal age, maternal anxiety and depression, maternal education, parental socioeconomic position, child IQ, parental conflict, presence of the child’s father, number of people living in the child’s home, bullying and family television use in early life. Each model was further adjusted for time spent engaging in one other activity on weekdays and weekends (time spent exercising [model 4b], time spent in a car/bus/train [model 4c], time spent outdoors in summer [model 4d], time spent playing outdoors in winter [model 4e], time spent playing with others [model 4f], time spent drawing, making or constructing things [model 4g], time spent on school or college work [model 4h], time spent reading [model 4i], time spent playing musical instruments [model 4j], time spent talking on a mobile phone [model 4k] and time spent talking on a landline phone [model 4l]. | | | | | | | |

**Table S7. Odds ratios for associations between depression and texting for models 4b-4l (100 multiply imputed datasets; n=14,665)**

|  | | Week days | | | Weekend days | | |
| --- | --- | --- | --- | --- | --- | --- | --- |
| **Model** | Hours of use | OR | 95%CI | p-value | OR | 95%CI | p-value |
| 4b | <1  1-2  3+ | 1.00  1.05  1.01 | 0.90, 1.21  0.82, 1.24 | 0.83 | 1.00  1.02  1.02 | 0.87, 1.20  0.84, 1.23 | 0.81 |
| 4c | <1  1-2  3+ | 1.00  1.06  1.02 | 0.91, 1.23  0.83, 1.25 | 0.77 | 1.00  1.03  1.03 | 0.88, 1.20  0.84, 1.25 | 0.76 |
| 4d | <1  1-2  3+ | 1.00  1.06  1.02 | 0.91, 1.23  0.83, 1.26 | 0.73 | 1.00  1.04  1.04 | 0.88, 1.21  0.85, 1.26 | 0.67 |
| 4e | <1  1-2  3+ | 1.00  1.05  1.01 | 0.91, 1.22  0.82, 1.24 | 0.84 | 1.00  1.03  1.03 | 0.88, 1.21  0.85, 1.24 | 0.74 |
| 4f | <1  1-2  3+ | 1.00  1.05  1.00 | 0.90, 1.21  0.82, 1.23 | 0.88 | 1.00  1.04  1.05 | 0.88, 1.22  0.86, 1.28 | 0.58 |
| 4g | <1  1-2  3+ | 1.00  1.05  1.01 | 0.91, 1.22  0.82, 1.23 | 0.86 | 1.00  1.03  1.03 | 0.88, 1.21  0.85, 1.24 | 0.73 |
| 4h | <1  1-2  3+ | 1.00  1.05  0.99 | 0.91, 1.22  0.81, 1.21 | 0.97 | 1.00  1.03  1.02 | 0.88, 1.20  0.84, 1.23 | 0.82 |
| 4i | <1  1-2  3+ | 1.00  1.05  1.02 | 0.91, 1.22  0.84, 1.25 | 0.74 | 1.00  1.03  1.04 | 0.88, 1.21  0.86, 1.26 | 0.64 |
| 4j | <1  1-2  3+ | 1.00  1.05  1.02 | 0.91, 1.22  0.83, 1.24 | 0.77 | 1.00  1.03  1.04 | 0.88, 1.21  0.86, 1.25 | 0.66 |
| 4k | <1  1-2  3+ | 1.00  1.05  1.02 | 0.90, 1.23  0.84, 1.25 | 0.73 | 1.00  1.06  1.09 | 0.91, 1.25  0.87, 1.35 | 0.42 |
| 4l | <1  1-2  3+ | 1.00  1.05  1.00 | 0.90, 1.21  0.82, 1.23 | 0.87 | 1.00  1.02  1.02 | 0.87, 1.20  0.84, 1.23 | 0.82 |
| Models 4b-4l adjusted for sex, previous depression (at age 15 years), maternal age, maternal anxiety and depression, maternal education, parental socioeconomic position, child IQ, parental conflict, presence of the child’s father, number of people living in the child’s home, bullying and family television use in early life. Each model was further adjusted for time spent engaging in one other activity on weekdays and weekends (time spent exercising [model 4b], time spent in a car/bus/train [model 4c], time spent outdoors in summer [model 4d], time spent playing outdoors in winter [model 4e], time spent playing with others [model 4f], time spent drawing, making or constructing things [model 4g], time spent on school or college work [model 4h], time spent reading [model 4i], time spent playing musical instruments [model 4j], time spent talking on a mobile phone [model 4k] and time spent talking on a landline phone [model 4l]. | | | | | | | |

**Table S8. Odds ratios for associations between anxiety and watching television, computer use and texting (complete case analysis; n=1,869).**

|  | | Week days | | | Weekend days | | |
| --- | --- | --- | --- | --- | --- | --- | --- |
| **Model** | Hours of use | OR | 95%CI | p-value | OR | 95%CI | p-value |
| Television |  |  |  |  |  |  |  |
| 1 | <1  1-2  3+ | 1.00  0.90  1.01 | 0.73, 1.11  0.78, 1.32 | 0.96 | 1.00  1.20  1.15 | 0.95, 1.53  0.89, 1.59 | 0.38 |
| 2 | <1  1-2  3+ | 1.00  0.97  1.05 | 0.78, 1.21  0.78, 1.39 | 0.79 | 1.00  1.27  1.17 | 0.99, 1.64  0.90, 1.53 | 0.38 |
| 3 | <1  1-2  3+ | 1.00  0.98  1.10 | 0.78, 1.22  0.82, 1.46 | 0.59 | 1.00  1.27  1.18 | 0.99, 1.63  0.90, 1.55 | 0.33 |
| 4g | <1  1-2  3+ | 1.00  0.98  1.06 | 0.79, 1.23  0.79, 1.41 | 0.75 | 1.00  1.24  1.10 | 0.96, 1.60  0.84, 1.45 | 0.57 |
| Computer use | |  |  |  |  |  |  |
| 1 | <1  1-2  3+ | 1.00  1.20  1.19 | 0.96, 1.50  0.93, 1.53 | 0.20 | 1.00  1.06  1.20 | 0.83, 1.34  0.94, 1.53 | 0.11 |
| 2 | <1  1-2  3+ | 1.00  1.29  1.34 | 1.01, 1.63  1.03, 1.75 | 0.04 | 1.00  1.20  1.37 | 0.93, 1.54  1.06, 1.77 | 0.02 |
| 3 | <1  1-2  3+ | 1.00  1.25  1.29 | 0.99, 1.59  0.99, 1.69 | 0.07 | 1.00  1.19  1.32 | 0.92, 1.53  1.02, 1.71 | 0.04 |
| 4g | <1  1-2  3+ | 1.00  1.19  1.12 | 0.93, 1.51  0.85, 1.47 | 0.50 | 1.00  1.14  1.18 | 0.88, 1.46  0.91, 1.54 | 0.22 |
| Texting |  |  |  |  |  |  |  |
| 1 | <1  1-2  3+ | 1.00  1.02  1.02 | 0.82, 1.27  0.79, 1.32 | 0.87 | 1.00  1.01  1.16 | 0.82, 1.26  0.92, 1.48 | 0.25 |
| 2 | <1  1-2  3+ | 1.00  0.90  0.82 | 0.72, 1.14  0.62, 1.09 | 0.13 | 1.00  0.89  0.95 | 0.71, 1.12  0.74, 1.23 | 0.53 |
| 3 | <1  1-2  3+ | 1.00  0.92  0.86 | 0.73, 1.16  0.65, 1.14 | 0.24 | 1.00  0.92  0.98 | 0.73, 1.16  0.76, 1.27 | 0.74 |
| 4a | <1  1-2  3+ | 1.00  0.92  0.85 | 0.73, 1.16  0.64, 1.13 | 0.22 | 1.00  0.94  1.01 | 0.74, 1.18  0.78, 1.31 | 0.77 |
| Model 1 was unadjusted.  Model 2 adjusted for sex, previous anxiety (at 15 years), maternal age, maternal anxiety and depression, maternal education, parental socioeconomic position.  Model 3 also adjusted for child IQ, parental conflict, presence of the child’s father, number of people living in the child’s home, bullying and family TV use in early life.  Model 4a further adjusted for time spent alone (weekdays or weekends, as applicable) | | | | | | | |

**Table S9. Odds ratios for associations between depression and watching television, computer use and texting (complete case analysis; n=1,869).**

|  | | Week days | | | Weekend days | | |
| --- | --- | --- | --- | --- | --- | --- | --- |
| **Model** | Hours of use | OR | 95%CI | p-value | OR | 95%CI | p-value |
| Television |  |  |  |  |  |  |  |
| 1 | <1  1-2  3+ | 1.00  0.99  1.27 | 0.80, 1.24  0.96, 1.67 | 0.12 | 1.00  1.19  1.22 | 0.93, 1.53  0.94, 1.60 | 0.17 |
| 2 | <1  1-2  3+ | 1.00  1.06  1.31 | 0.84, 1.33  0.98, 1.74 | 0.07 | 1.00  1.25  1.23 | 0.96, 1.61  0.93, 1.63 | 0.09 |
| 3 | <1  1-2  3+ | 1.00  1.07  1.35 | 0.85, 1.35  1.01, 1.81 | 0.05 | 1.00  1.23  1.23 | 0.95, 1.60  0.93, 1.63 | 0.11 |
| 4g | <1  1-2  3+ | 1.00  1.07  1.34 | 0.84, 1.35  1.00, 1.79 | 0.06 | 1.00  1.23  1.21 | 0.94, 1.59  0.91, 1.60 | 0.15 |
| Computer use | |  |  |  |  |  |  |
| 1 | <1  1-2  3+ | 1.00  1.05  1.08 | 0.83, 1.32  0.83, 1.40 | 0.57 | 1.00  1.02  1.30 | 0.80, 1.31  1.01, 1.68 | 0.02 |
| 2 | <1  1-2  3+ | 1.00  1.12  1.20 | 0.88, 1.43  0.91, 1.57 | 0.17 | 1.00  1.16  1.53 | 0.90, 1.50  1.18, 1.99 | 0.001 |
| 3 | <1  1-2  3+ | 1.00  1.10  1.16 | 0.87, 1.41  0.88, 1.52 | 0.27 | 1.00  1.15  1.50 | 0.89, 1.50  1.15, 1.95 | 0.001 |
| 4g | <1  1-2  3+ | 1.00  1.09  1.11 | 0.85, 1.39  0.84, 1.47 | 0.46 | 1.00  1.14  1.47 | 0.88, 1.49  1.12, 1.93 | 0.003 |
| Texting |  |  |  |  |  |  |  |
| 1 | <1  1-2  3+ | 1.00  1.29  1.27 | 1.03, 1.62  0.98, 1.65 | 0.02 | 1.00  1.23  1.26 | 0.99, 1.54  0.99, 1.61 | 0.03 |
| 2 | <1  1-2  3+ | 1.00  1.17  1.02 | 0.92, 1.48  0.78, 1.35 | 0.59 | 1.00  1.10  1.01 | 0.87, 1.38  0.78, 1.32 | 0.81 |
| 3 | <1  1-2  3+ | 1.00  1.18  1.03 | 0.93, 1.49  0.78, 1.37 | 0.58 | 1.00  1.11  1.02 | 0.88, 1.40  0.78, 1.32 | 0.84 |
| 4a | <1  1-2  3+ | 1.00  1.18  1.03 | 0.93, 1.49  0.78, 1.37 | 0.60 | 1.00  1.12  1.02 | 0.88, 1.41  0.79, 1.33 | 0.84 |
| Model 1 was unadjusted.  Model 2 adjusted for sex, previous depression (at 15 years), maternal age, maternal anxiety and depression, maternal education, parental socioeconomic position.  Model 3 also adjusted for child IQ, parental conflict, presence of the child’s father, number of people living in the child’s home, bullying and family TV use in early life.  Model 4a further adjusted for time spent alone (weekdays or weekends, as applicable) | | | | | | | |

**Table S10. Odds ratios for associations between anxiety and depression and watching television, computer use and texting when imputed values of anxiety/depression were re-categorised**

|  | | Week days | | | Weekend days | | |  |
| --- | --- | --- | --- | --- | --- | --- | --- | --- |
| **Model** | Hours of use | OR | 95%CI | p-value | OR | 95%CI | p-value |  |
| Anxiety |  |  |  |  |  |  |  |  |
| Television |  |  |  |  |  |  |  |  |
| 3 | <1  1-2  3+ | 1.00  0.99  1.09 | 0.88, 1.12  0.94, 1.28 | 0.25 | 1.00  1.01  1.05 | 0.89, 1.18  0.90, 1.22 | 0.50 |  |
| Computer use | |  |  |  |  |  |  |  |
| 3 | <1  1-2  3+ | 1.00  1.09  1.19 | 0.96, 1.24  1.04, 1.38 | 0.02 | 1.00  1.09  1.16 | 0.94, 1.26  1.00, 1.34 | 0.06 |  |
| Texting |  |  |  |  |  |  |  |  |
| 3 | <1  1-2  3+ | 1.00  1.02  1.04 | 0.89, 1.16  0.86, 1.26 | 0.66 | 1.00  0.97  1.08 | 0.84, 1.12  0.89, 1.30 | 0.52 |  |
| Depression | |  |  |  |  |  |  |  |
| Television |  |  |  |  |  |  |  |  |
| 3 | <1  1-2  3+ | 1.00  0.98  1.13 | 0.84, 1.13  0.94, 1.35 | 0.19 | 1.00  0.94  1.07 | 0.80, 1.10  0.90, 1.28 | 0.30 |  |
| Computer Use | |  |  |  |  |  |  |  |
| 3 | <1  1-2  3+ | 1.00  1.00  1.08 | 0.87, 1.15  0.90, 1.28 | 0.38 | 1.00  1.06  1.20 | 0.92, 1.22  1.04, 1.39 | 0.01 |  |
| Texting |  |  |  |  |  |  |  |  |
| 3 | <1  1-2  3+ | 1.00  1.05  1.06 | 0.94, 1.17  0.91, 1.24 | 0.36 | 1.00  1.05  1.08 | 0.93, 1.19  0.93, 1.25 | 0.26 |  |
| Model 3 adjusted for sex, previous anxiety/depression (at 15 years), maternal age, maternal anxiety and depression, maternal education, parental socioeconomic position, adjusted for child IQ, parental conflict, presence of the child’s father, number of people living in the child’s home, bullying and family TV use in early life.  In this sensitivity analysis imputed values of depression and anxiety were re-categorised as only level higher than predicted by the imputation model (unless they were already predicted as being in the highest category) | | | | | | | |  |
